# Discussion on Local Spark Sintering of a Ceramic-Metal System in an SR-CT Experiment during Microwave Processing

**DOI:** 10.3390/ma9030132

**Published:** 2016-02-26

**Authors:** Yongcun Li, Feng Xu, Xiaofang Hu, Bo Dong, Yunbo Luan, Yu Xiao

**Affiliations:** 1 College of Mechanics, Taiyuan University of Technology, Taiyuan 030024, China; liyongcun@tyut.edu.cn (Y.L.); luanyunbo@tyut.edu.cn (Y.L.); 2 CAS Key Laboratory of Mechanical Behavior and Design of Materials, Department of Modern Mechanics, University of Science and Technology of China, Hefei 230026, China; huxf@ustc.edu.cn (X.H.); dongbo@mail.ustc.edu.cn (B.D.); xiaoyuxy@mail.ustc.edu.cn (Y.X.)

**Keywords:** microwave sintering, microstructureevolution, metal, synchrotron radiation computed tomography

## Abstract

In this paper, local spark sintering of a ceramic-metal system (SiO_2_-Sn) during microwave processing was examinedby means of synchrotron-radiation-computed tomography technology. From the reconstructed 3-D and cross-section images of the specimen, adensification process was observed below the melting point of Sn, and then the specimen came into a rapid densification stage. These results may be due to the local spark sintering induced by the high-frequency alternating microwave electric fields. As the metallic particles Sn were introduced, the microstructure of “ceramic-metal” will lead to a non-uniform distribution and micro-focusing effect from electric fields in some regions (e.g., the neck). This will result in high-intensity electric fields and then induce rapid spark sintering within the micro-region. However, in the subsequent stage, the densification rate declined even when the specimen was not dense enough. The explanation for this is that as the liquid Sn permeated the gaps between SiO_2_, the specimen became dense and the micro-focusing effect of electric fields decreased. This may result in the decrease or disappearance of spark sintering. These results will contribute to the understanding of microwave sintering mechanisms and the improvement of microwave processing methods.

## 1. Introduction

Over the last decades, microwave sintering has been under constant development for the rapid preparation of high-performance powder materials, such as ceramics and ceramic matrix composites [1,2,3,4,5]. Recently, since the first full sintering of metal powders in a microwave field, many experiments on microwave sintering of various kinds of metals, including ceramic-metal materials, have been carried out. For example, E. Breval *et al.* [6] indicated that in the microwave sintering of WC-Co, there was very little WC particle growth and the specimen possessed six times more resistance against corrosion and a hardness 1–5 GPa higher than the conventional specimens.

Thus far, much work has been performed to study the mechanisms of microwave sintering. Many researchers attribute the advantages of microwave sintering to the effect induced byhigh-frequency alternating microwave fields, such as the enhancement of the diffusion coefficient [7,8], reduction of activation energy [9,10], micro-focusing effect [11] and the eddy current [12] for ceramics or metals. However, as for the metal-ceramic materials, due to the role of high-energy microwave fields, as well as the heterogeneity and non-uniform distribution of the mixed materials, there might besome special interaction mechanisms that are different from the microwave sinteringof pure ceramicand metal. For example, as the WC and Co mixed together, the heating efficiency of WC/Co in the magnetic-field strangely became lower than both the WC and Co [13]. These results indicate thattheinteraction mechanisms of themixed systemarenot just a simple superposition of the original mechanisms. These mechanisms may affect the microstructure and macro-performance of materials. This means it is quite necessary to explore the sintering mechanisms in the microwave sintering of metal-ceramic materials.

At present, the technology of TEM, SEM, hot-stage microscopes, *etc*. are usually adopted to study microwave sintering mechanisms [12,14]. These methods can be usedtocarry out the *in* *situ* analysis of the surface or slice information of a specimen. However, owing to the hightemperature, microwave radiation and the opacity of materials, it is difficult to realize theinternal and real-time microstructure evolution observation continuously during microwave processing. The synchrotron radiation computer tomography (SR-CT) technique is the latest non-destructive testing technology [15] based on the excellent synchrotron radiation light source (e.g., high intensity, strong penetrability and good coherence). It can achieve internal and real-time microstructure evolution observation of materials under extreme conditions (e.g., high temperature, intense radiation). By applying this technique, the surface and internal microstructure evolution during high-temperature microwave processing can be directly and continuously observed.

In this paper, the SR-CT technique was adopted toinvestigate the microstructure evolution of ceramic-metal system (silicon dioxide and tin, SiO_2_-Sn) during microwave sintering. In theexperiment, 3-D and cross-section images of the same microstructureat differenttimes were obtained, and some typical sintering phenomena were clearly observed. Also, a densification processwas observed below the melting point of Sn, and then the specimen came into a rapid densification stage. Particle rotation and rearrangement was frequently observed. However, in the subsequent stage, the densification rate declined even when the specimen was not dense enough. The reason may be due to the decrease or disappearance ofspark sintering. As the liquid Sn permeated the gaps between particles, the specimen became dense and themicro-focusing effect of microwave electric fields decreased as a result. These results will contribute to the understanding of the rapid microwavesintering mechanisms of materials and the improvement of microwave processing methods.

## 2. Materials and methods

### 2.1. Materials

In our experiment, chemically pure SiO_2_ (purity 99.9%, average diameter 150 μm) and Sn (purity 99.8%, average diameter 75 μm) powders were used. Before theexperiment, SiO_2_-Sn powders (volume ratio of 1:10) were mixed uniformly in the anhydrous ethanol by the mechanical agitator for 4 h, then dried in the vacuum drying oven and loosely encapsulated into a closed quartz capillary (height: 10 mm, internal radius: 0.35 mm).

### 2.2. The SR-CT Experiment during Microwave Processing

**Figure 1:**
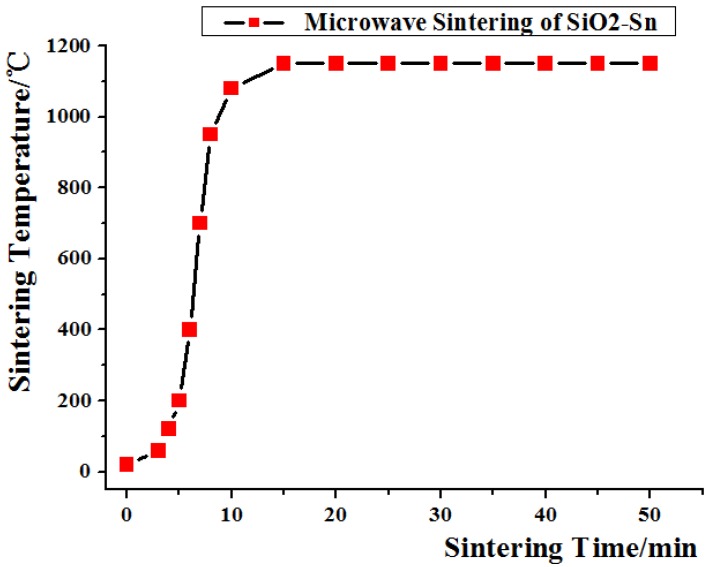
Typical temperature profile of SiO_2_-Sn during microwave sintering.

The SR-CT experiment on microwave sinteringof SiO_2_-Sn was carried out on the BL13W1 beam line at the Shanghai Synchrotron Radiation Facility (SSRF, Shanghai, China). In the experiment, the specimen was introduced into a specially designed microwave furnace (multimode cavity: 2.45 GHz, output power: 3 kW). The sintering temperature wasmeasured by a thermo tracer [type TH5104, range: 10–1500 °C, accuracy ± 1.0% (full scale)], and the typical temperature profile is shown in Figure 1.The temperature was compared with and calibrated by the thermocouple. Other goodresearch on calibration of temperature during the microwave sintering process is available elsewhere [16]. In the experiment, marking points (Cu particleswith a radius of about 20 μm were affixed on the capillary surface as marking points) were used to roughly track the same part of the sample, which usually contains several cross-sections. After finding the same part of the sample at different sintering times, one of the cross-sections images (gray images) within this part of the sample can be selected, and then the related algorithm can be employed to identify this cross-section with other cross-sections of the sample at different sintering times. Two cross-sections were considered the same cross-sections of this part of the sample when the correlation coefficient between these two figures reached the largest value.

## 3. Results and Discussion

**Figure 2:**
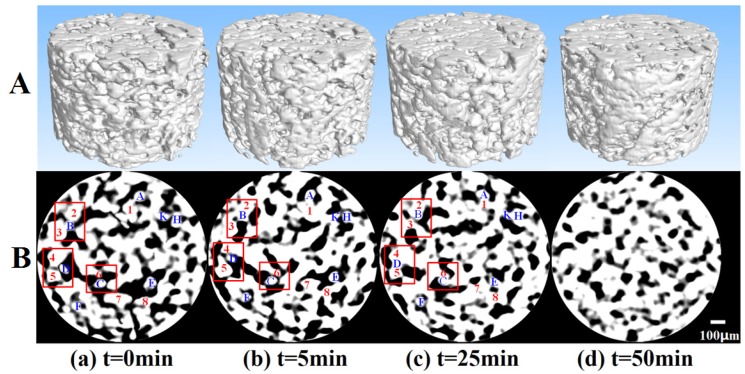
3-Dand cross-section images of the evolution of the same the same cross-sectionat different sintering times.

Figure 2 shows the microstructure evolution of the same 3-D and cross-section of SiO_2_-Sn atdifferent sintering times. Grayscale range is from 0 to 255; the closer to 255, the higher the relative density, which means that white represents particles and black represents pores. In Figure 2B, some typical particles were marked with numbers and letters (particles in blue color represent Sn, and red color represents SiO_2_). From these images, the evolution of the same microstructurecan be tracked. For example, after microwave processing for some minutes, most of separated particles in Figure 2B(a) connected with each other and sinteringnecks formed in Figure 2B(b). The pores were interconnected with each other in Figure 2B(a), while in Figure 2B(d) the shape of pores changed and became closed and isolated. This means the specimen gradually changed from loose to dense. These phenomena were in accordance with the sintering theory and can also be observed insolid microwave sintering (e.g., Al-SiC, [17]). In addition, there were some special sintering phenomena. For example, the microstructure evolution can be well tracked during solid microwave sintering of Al-SiC [17]. However, in the microwave sintering of SiO_2_-Sn, the rearrangement and rotation of particles were more frequent, and it wasvery hard to track the same microstructure during sintering. As we know, in microwave sintering the heat is derived from the direct interaction between microwave and material. This means the heating characteristic within the material is inevitably related to the microstructure and itsmaterial evolution. In order to investigate microwave sintering mechanisms, quantitative analysis of microstructure evolution parameters is an effective and important method.

**Figure 3:**
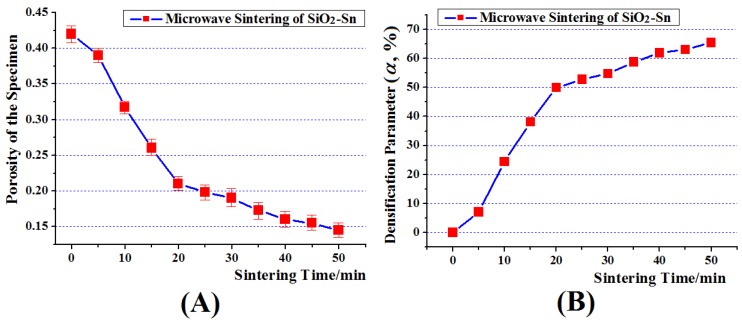
(**A**) The relationship between porosity and sintering time; and (**B**) the relationship between densification parameter and sintering time.

In this section, the statistics of the porosity of specimen in the differenttimes were carried out. The porosity represents the local bulk percentage of pore in the specimen. It is the averageof several similar cross-sections. The results are shown in Figure 3A. In the sintering theory, the densification parameter αis usually adopted to analyze the densification process of sintering. It can be described as follows,

(1) $$\alpha =\frac{\rho -\rho_{0}}{\rho_{1}-\rho_{0}}\times 100\%$$

Here, ρ represents true density, and $\rho_{0}$ and $\rho_{1}$ representthe density of the green body and theoretical density, respectively. This means the densification parameter α can be described as

(2) $$\alpha =\frac{\rho /\rho_{1}-\rho_{0}/\rho_{1}}{1-\rho_{0}/\rho_{1}}\times 100\%=\frac{（1-\beta ）-（1-\beta_{0}）}{1-（1-\beta_{0}）}\times 100\%=\frac{\beta_{0}-\beta }{\beta_{0}}\times 100\%$$

Here β represents true porosity, and $\beta_{0}$ represents the porosity of the green body. Inorder to study the densification process of SiO_2_-Sn during microwave sintering, the relationship between α and sintering time is shown in Figure 3B. In Section 3.1, the densification process and sintering mechanisms ofthe microstructure evolutionof the specimen will be discussed.

### 3.1. Densification below the Melting Point of Sn Induced by Microwave E-Fields

Figure 3B shows the change in α at different sintering times. It can be seen that the densification rate was very largefrom the 5th to 20th minute, and declined gradually from the 20th to 50th minute. Moreover, at the beginning, the value of α began to rise from 0 to 7.08 in the first 5 minute. However, the highest sintering temperature during this period was only 200 °C, which was below the melting point of Sn (231 °C). Although the sintering would occur between particles of Sn at this temperature, the volume ratio of Sn is only 1/11, and most Sn particles were separated by SiO_2_ particles. How did the decline of porosity occur?

**Figure 4:**
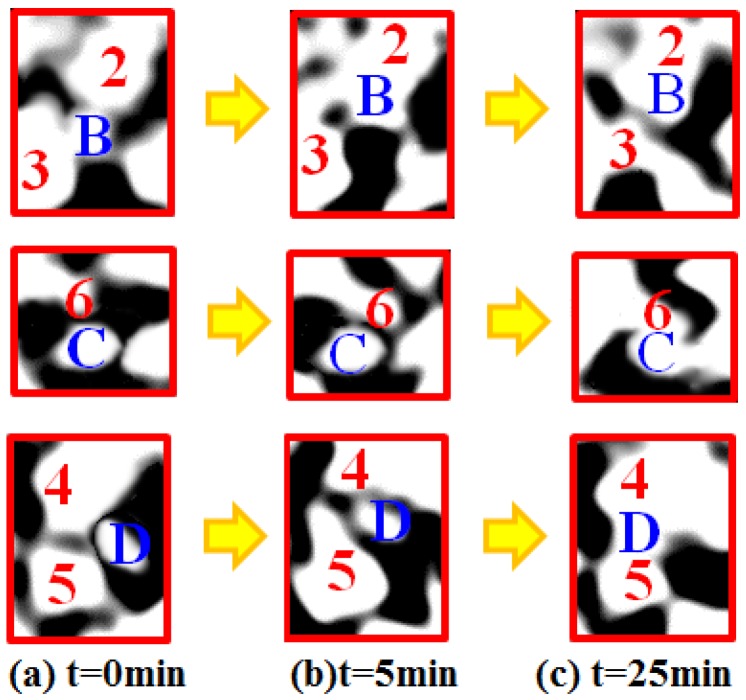
The local amplification images of particles B, C and D in Figure 2B.

Figure 3 shows that the main densification began to occurin the first 5 minute. The corresponding microstructure evolution during this period is shown in Figure 2B(a,b). From these images, the microstructure evolution can be clearly observed. For example, particle B was separated from particles 2 and 3 at the beginning. However, until the 5th minute, particle B was sintered together with particles 2 and 3. The local amplification images of these particles are shown in Figure 4a similar microstructure evolution phenomenon was also observed on the other cross-section of the sample. This will inevitably lead to the densification of specimen.This phenomenon may be induced by the spark sintering. The explanation is that, as Figure 4b shows, the sintering has happened between particles B, 2 and 3. This rapid sintering in such a short time and below the melting point of Sn indicates that there was a lot of heat deposition between particles induced by microwave fields, which then led to the high temperature within the local connection regions between particles. This highly energy deposition and high temperature is most likely due to the spark sintering caused by microwave electric fields.

As we know, spark sintering is a rapid sintering method viaelectric spark between particles induced by current. Yet in microwave sintering, the microwave energy is the direct energy source for the sintering process. This meansspark sintering likely must be closely related to the intensity distribution of microwave E-fields. In order to further study the reasons for spark sintering, it is necessary to research the intensity distribution of microwave E-fields within the specimen.

To study the intensity distribution of microwave E-fields, the TE101 mode resonator (109.2 mm × 54.6 mm × 74 mm) was used. With this resonator, the distribution of microwave electric fieldwas analysed by using the HFSS finite element analysis software. The TE101 mode resonator is a single-mode microwave resonator. It sets a 1 and 1 standing wave cycle of the electric fields in the direction of the x and z axes, respectively, and sets the electric field to be uniform and parallel to the *y* axis. In this way, an investigation into the propagation characteristics of microwave E-fields in a certain direction can be performed by using this resonator. To compare the obtained results with those obtained experimentally, the microwave frequency is set to 2.45 GHz, and the solution type is set to driven modal. The ceramic particle of SiO_2_ with a diameter of 150 μm and metallic particle Sn with a diameter of 75 μm is selected as the material model, which is identical to that used experimentally.Using this model, the E-field distribution between two of the connected particles was examined. Appropriate relative permittivity and relative magnetic permeability of 9.8 and 1.0, respectively, are assigned to the ceramic particles of SiO_2_. The appropriate relative permittivity and relative magnetic permeability of 1.0 and 1.0, respectively, are assigned to the metallic particle of Sn. Note that other values can be chosen for these two parameters if other materials are simulated. To investigate the behaviour of the microwave E-fields independently, the particles were placed in the region where the E-fields are at a maximum and where the H-fields are almost zero.

**Figure 5:**
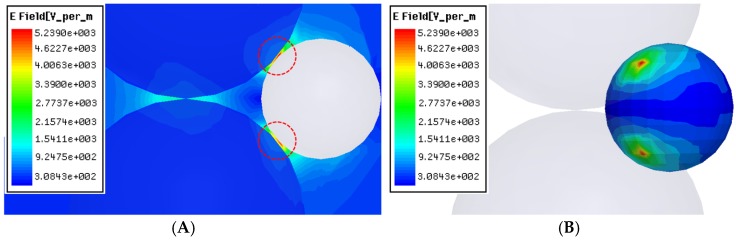
The intensity distribution of microwave E-fields (**A**) within the contacted particles of SiO_2_-Sn; and (**B**) on the surface of particle Sn.

In the TE101 rectangular single-mode cavity, an operating frequency of 2.45 GHz will produce a wavelength around 122.5 mm, and the half wavelength of the standing wave are 109.2mm and 74mm in the direction of the x and z axes, respectively. Comparing the size of the particles (diameter 0.075 mm) with the half wavelength of the standing wave, the microwave E-fields in the sample can be considered to be approximately homogeneous prior to the introduction of the particles. Figure 5 shows the intensity distribution of microwave E-fields when the particle Sn is located within the sintering neck of SiO_2_, which is similar to the case of particles B, C and D located within the sintering neck of SiO_2_ particles 2 to 6.

As can be seen, the E-fields within the micro-region of sintering neck between SiO_2_ are much larger than the average fields, which suggests that the E-fields are focused in this micro-region. However, it can also be seen that the E-fields within the micro-region of the sintering neck between SiO_2_-Sn are much larger than even the E-fields between SiO_2_, which indicates that the focusing effect of E-fields is much more significantin this local micro-region.In the simulation, we assume that the intensity of the applied field is 3.30 × 10^2^ v/m. Then it can be determined that the peak E-field between SiO_2_-Sn within the neck region is 5.24 × 10^3^ v/m, which is about 15.88 times larger than the applied field. In the actual sintering, the intensity of the applied field will be very high, so the peak E-field within the neck region between SiO_2_-Sn will be much higher. For example, if the average E-field during microwave sintering is 1 kV/cm (e.g., ZnO [11]), the peak E-fields can be as high as 15.88 kV/cm. Because the square amplitude of the electric field *E^2^* is directly related to the power density of microwaves [11], and thus the heating rate, the peak microwave energy is approximately 250 times greater than the applied microwave energy at the beginning of the sintering process, which may be large enough to cause microscopic ionization at atmospheric pressure and lead to spark sintering [11,18,19]. Therefore, although the applied field is not very high and the overall sample temperature is below the melting point of Sn, the peak E-fields and temperature within the micro-region between SiO_2_-Sn will be high enough and induce local spark sintering. As a result, mass diffusion will be increased and the sintering process will be accelerated as well. This factor may be an important mechanism for rapid preparation involving microwave sintering.

### 3.2. Rapid Densification of the Specimen in the Middle Sintering Period

As the sintering process wenton, the sintering temperature increasedrapidly (e.g., by the 10th minute the temperature reached 1080 °C)and thedensification rate became quite large. As shown in Figure 2B, particles C and D were still separated fromparticles 6 and 4, 5 at the 5th minute. However, in the 25th minute these particles were sintered together.The local amplification images of particles B, C and D in Figure 2B are shown in Figure 4.

**Figure 6:**
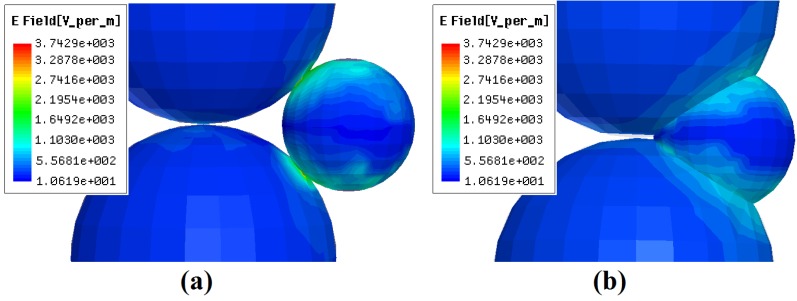
The intensity distribution of microwave E-fields in the particles of SiO_2_-Sn at different sintering stages (**a**) the sintering neck is 0.12 times of the average of the radius of Sn and SiO_2_; and (**b**) the distance between the centers of Sn and SiO_2_ is 0.75 times of the sum of the radius of Sn and SiO_2_.

This phenomenon may also be due to thespark sintering, because there was initially a distance between particles C and 6, as well as between particles D and 4, 5. As the sintering process wenton, the particles Sn entered aliquid phase and the sample started to shrink. This may make these particles contact each other. This means spark sintering may occur, induced by the micro-focusing effect of microwave E-fields between these particles. This is the same as the case that happened between particles B and 2, 3 in the first 5 minutes. Besides, as the sintering neck between particles grew larger, there was still a focusing effect between particles. Figure 6a shows the distribution of microwave E-fields on the particle surface when the sinteringneck is 0.12 times the average of the radius of Sn and SiO_2_ [there was still a pore between the particles of SiO_2_-Sn and the sintering state is between the sintering states of Figure 4b,c]. From Figure 6a, it can be seen that there is still a focusing effect within the micro-region of the sintering neck between SiO_2_-Sn. Here, the peak E-field between SiO_2_-Sn within the neck region is 3.74×10^3^ v/m, which is about 11.33 times larger than the applied field (3.30×10^2^ v/m).This means that in the actual sintering, the intensity of the peak electric field within the neck region between SiO_2_-Sn will be 11.33 times larger than the applied fields, and the peak *E*^2^ field is approximately 128 times greater than the applied *E*^2^ field, which may also cause microscopic ionization and lead to spark sintering.

### 3.3. Decline of Densification Rate when the Specimen Was not Dense Enough

Section 3.1 has discussed the rapid densification process of SiO_2_-Sn during microwave sintering from the 5th to 25th minute. However, from the 20th minute, the densification rate declined a lot (as shown in Figure 3B). This phenomenon also happens in the conventional liquid sintering, and usually happens when the densification parameter reaches about 75~80 [20]. However, in this study the densification parameter of the specimen during this period was only 50~65. This means that there were still considerable pores and the specimen was not dense enough, like the image shown in Figure 2B(d). There are some reasons for this phenomenon: Firstly,the highest sintering temperature is about 1150°C, which is a little lower than the usually sintering temperature of SiO_2_ (>1200 °C). This means thatin the laterstage, the solid sintering rate between SiO_2_ particles would be blocked because of the lower temperature. Secondly, due to the small volume fraction of Sn (10%), as the specimen became densethe distance between particles decreased at the same time, and the viscous flow of Sn and particle rearrangement would decline gradually.Besides, the decrease of the distance between particles will make the friction between particles increases, which will inhibit the further densification ofthe specimen. Thirdly, as the specimen became dense, the liquid phase of Sn penetrated the gap between particles. This will produce a change in microstructure configuration and may result in the reduction or disappearance of spark sintering. Figure 6b shows the distribution of microwave E-fields on the particle surface of SiO_2_-Sn (the distance between the centers of Sn-SiO_2_ is 0.75 times of the sum of the radius of Sn and SiO_2_). This sintering state is between the sintering states of Figure 4b,c. From this figure, it can be seen that the peak E-field between SiO_2_ and Sn within the neck region is 1.10 × 10^3^ v/m. This means that the intensity of peak E-fields within the neck region between SiO_2_ and Sn was only 3.33 times that of the applied fields, and cannot be large enough to cause microscopic ionization at atmospheric pressure and lead to spark sintering. This meansthe driving force for the mass diffusion decreased and the sintering process including the densification process declined as a result. These may be the reasons why the densification rate declined when the specimen was not dense enough.

## 4. Conclusions

An *in* *situ* investigation on local spark sintering of ceramic-metal system (SiO_2_-Sn) during microwave processing was carried out using synchrotron radiation computed tomography technology (SR-CT).The following conclusions can be drawn:

In the experiment, except for the normal sintering phenomenon, a densification process was observed below the melting point of Sn, and then the specimen came into a rapid densification stage. This result may be due to the spark sintering induced by the high-frequency alternating microwave electric fields.

In the ceramic-metal system (Sn-SiO_2_), as the metallic particles were introduced, the microstructure of “ceramic-metal” will lead to a non-uniform distribution and micro-focusing effectofelectric fields in some regions (e.g., the neck). This will result in high intensity electric fields and may induce the local rapid spark sintering within micro-region.

In the later sintering stage, as the liquid Sn permeated the gaps between SiO_2_, the specimen became dense and the micro-focusing effect ofelectric fields decreased. This may result in the decrease or disappearance of spark sintering, so the densification rate declined even when the specimen was not dense enough.

These mechanisms may be the explanation for the microstructure evolution process during microwave sintering of SiO_2_-Sn, and may contribute to the understanding of microwave sintering mechanisms and the improvement of microwave processing methods.

## References

[1] Yadoji P., Peelamedu R., Agrawal D., Roy R. (2003). Microwave sintering of Ni–Zn ferrites: Comparison with conventional sintering. Mat. Sci. Eng. B.

[2] Agrawal D. (2010). Latest global developments in microwave materials processing. Mater. Res. Innov..

[3] Clark D.E., Folz D.C., West J.K. (2000). Processing materials with microwave energy. Mat. Sci. Eng. A.

[4] Morteza O., Omid M. (2010). Microwave versus conventional sintering: A review of fundamentals, advantages and applications. J. Alloy. Compd..

[5] Cheng J., Agrawal D., Zhang Y., Roy R. (2002). Microwave sintering of transparent alumina. Mater. Lett..

[6] Breval E., Cheng J.P., Agrawal D.K., Gigl P., Dennisb M., Roya R., Papworth A.J. (2005). Comparison between microwave and conventional sintering of WC/Co composites. Mat. Sci. Eng. A.

[7] Wilson B.A., Lee K.Y., Case E.D. (1997). Diffusive crack-healing behavior in polycrystalline alumina: A comparison between microwave annealing and conventional annealing. Mater. Res. Bull..

[8] Janney M.A., Kimrey H.D., Schmidt M.A., Kiggans J.O. (1991). Grain growth in microwave-annealed alumina. J. Am. Ceram. Soc..

[9] Janney M.A., Kimrey H.D., Allen W.R., Kiggans J.O. (1997). Enhanced diffusion in sapphire during microwave heating. J. Mater. Sci..

[10] Demirskyi D., Agrawal D., Ragulya A. (2011). Neck growth kinetics during microwave sintering of nickel powder. J. Alloy. Compd..

[11] Birnboim A., Calame J.P., Carmel Y. (1999). Microfocusing and polarization effects in spherical neck ceramic microstructures during microwave processing. J. Appl. Phys..

[12] Ma J., Diehl J.F., Johnson E.J., Martin K.R., Miskovsky N.M., Smith C.T., Weisel G.J., Weiss B.L., Zimmerman D.T. (2007). Systematic study of microwave absorption, heating, and microstructure evolution of porous copper powder metal compacts. J. Appl. Phys..

[13] Cheng J.P., Roy R., Agrawal D. (2002). Radically different effects on materials by separated microwave. Mater. Res. Innov..

[14] Subhadip B., Susmita B., Bandyopadhyay A. (2011). Densification study and mechanical properties of microwave-sintered mullite and mullite–zirconia composites. J. Am. Ceram. Soc..

[15] Li X., Hu X.F. (1999). Synchrotron radiation tomography for reconstruction of layer structures and internal damage of composite material. Chin. J. Lasers B.

[16] Zuo F., Saunier S., Marinel S., Chanin-Lambert P., Peillon N., Goeuriot D. (2015). Investigation of the mechanism(s) controlling microwave sintering of α-alumina: Influence of the powder parameters on the grain growth, thermodynamics and densification kinetics. J. Eur. Ceram. Soc..

[17] Li Y.C., Xu F., Hu X.F., Kang D., Xiao T.P., Wu X.P. (2014). In situ investigation on the mixed-interaction mechanisms in the metal–ceramic system’s microwave sintering. Acta Mater..

[18] Su H., Lynn J.D. (1996). Sintering of Alumina in Microwave-Induced Oxygen Plasma. J. Am. Ceram. Soc..

[19] Nouari S., Zafar I., Abdullah K., Hakeem A.S., Nasser A.A., Tahar L., Amro A.Q., Kirchner R. (2013). Spark plasma sintering of metals and metal matrix nanocomposites: A review. J. Nanomater..

[20] Kingery W.D. (1959). Densification during sintering in the presence of a liquid phase. II.Experimental. J. Appl. Phys..

